# Alternative Splicing Changes Promoted by NOVA2 Upregulation in Endothelial Cells and Relevance for Gastric Cancer

**DOI:** 10.3390/ijms24098102

**Published:** 2023-04-30

**Authors:** Anna Di Matteo, Elisa Belloni, Davide Pradella, Anna Maria Chiaravalli, Giacomo Maria Pini, Mattia Bugatti, Roberta Alfieri, Chiara Barzan, Elena Franganillo Tena, Silvia Bione, Elisa Terenzani, Fausto Sessa, Christopher D. R. Wyatt, William Vermi, Claudia Ghigna

**Affiliations:** 1 Istituto di Genetica Molecolare “Luigi Luca Cavalli-Sforza”, Consiglio Nazionale delle Ricerche, 27100 Pavia, Italy; 2 Department of Pathology, Ospedale di Circolo, ASST-Sette Laghi, 21100 Varese, Italy; 3 Department of Molecular and Translational Medicine, University of Brescia, 25100 Brescia, Italy; 4 Istituto Universitario di Studi Superiori (IUSS), Università degli Studi di Pavia, 27100 Pavia, Italy; 5 Dipartimento di Biologia e Biotecnologie “Lazzaro Spallanzani”, Università degli Studi di Pavia, 27100 Pavia, Italy; 6 Department of Medicine and Surgery, Università degli Studi dell’Insubria, 21100 Varese, Italy; 7 Centre for Genomic Regulation, The Barcelona Institute of Science and Technology, 08036 Barcelona, Spain; 8 Department of Pathology and Immunology, Washington University School of Medicine, St. Louis, MO 63110-1010, USA

**Keywords:** alternative splicing, angiogenesis, tumor vasculature, gastric cancer, cancer biomarkers, RNA binding proteins

## Abstract

Angiogenesis is crucial for cancer progression. While several anti-angiogenic drugs are in use for cancer treatment, their clinical benefits are unsatisfactory. Thus, a deeper understanding of the mechanisms sustaining cancer vessel growth is fundamental to identify novel biomarkers and therapeutic targets. Alternative splicing (AS) is an essential modifier of human proteome diversity. Nevertheless, AS contribution to tumor vasculature development is poorly known. The Neuro-Oncological Ventral Antigen 2 (NOVA2) is a critical AS regulator of angiogenesis and vascular development. NOVA2 is upregulated in tumor endothelial cells (ECs) of different cancers, thus representing a potential driver of tumor blood vessel aberrancies. Here, we identified novel AS transcripts generated upon NOVA2 upregulation in ECs, suggesting a pervasive role of NOVA2 in vascular biology. In addition, we report that NOVA2 is also upregulated in ECs of gastric cancer (GC), and its expression correlates with poor overall survival of GC patients. Finally, we found that the AS of the *Rap Guanine Nucleotide Exchange Factor 6* (*RapGEF6*), a newly identified NOVA2 target, is altered in GC patients and associated with NOVA2 expression, tumor angiogenesis, and poor patient outcome. Our findings provide a better understanding of GC biology and suggest that AS might be exploited to identify novel biomarkers and therapeutics for anti-angiogenic GC treatments.

## 1. Introduction

Angiogenesis, the formation of new blood vessels from pre-existing vessels, is a crucial process for cancer progression and metastasis formation [1]. Consequently, anti-angiogenic treatments have been developed to block oxygen and nutrient supply to tumor cells, thus inducing cancer regression [2]. However, the clinical efficacy of these therapies is so far modest, and the emergence of resistance and compensatory mechanisms is a common event [2]. 

Tumor blood vessels differ in both morphology and functionality when compared with normal vessels [3]. For instance, tumor vessels are disorganized, dilated, tortuous, highly permeable, and characterized by loss of their hierarchy [3]. A better characterization of the molecular alterations sustaining tumor angiogenesis is fundamental to understanding the tumor vasculature’s aberrant phenotypes and implementing the efficacy of anti-angiogenic therapies.

Alternative splicing (AS) is the post-transcriptional mechanism through which a single primary transcript (pre-mRNA) generates multiple mature mRNAs, thus allowing the production of different protein variants from a single gene [4]. AS affects approximately 95% of human coding-protein genes [5,6] and it represents an essential regulator of gene expression, protein activity, and proteome diversity. Mechanistically, AS programs are orchestrated by splicing regulatory factors (SRFs) that interact with negative/positive *cis*-acting RNA motifs located within a regulated exon or in the adjacent introns, resulting in the inhibition/promotion of spliceosome assembly and AS event orchestration [4]. Highly coordinated splicing programs control key cellular processes during development, cell identity, and cell reprogramming or in response to environmental stimuli [7,8]. Therefore, it is not unexpected that AS dysregulation is causally linked to carcinogenesis [9,10,11]. Different SRFs that function as *bona fide* oncoproteins or tumor suppressors are commonly mutated or aberrantly expressed in cancer cells [12,13] resulting in mis-splicing events that affect tumor establishment, progression, and resistance to therapeutic treatments [10,11,14,15]. Given that AS dysregulation acts as an important molecular modifier of oncogenesis, it is considered a hallmark of cancer [10,11,16]. Remarkably, a large fraction of AS isoforms expressed in tumors are cancer-specific and not detectable in healthy tissues [17,18,19,20]. Besides providing an opportunity for early disease detection and patient stratification, cancer-restricted AS variants are also exploitable to develop more effective therapeutic approaches [10,14]. Although the role of aberrant AS regulation in cancer cells is well established [10,11], its effective contribution to the development of tumor vasculature is poorly known, thus limiting the possibility of identifying novel specific targets for anti-angiogenic therapy. NOVA2 is a tissue-restricted SRF that, for a long time, has been considered specific to neural cells of the central nervous system (CNS), where it plays a crucial role in neuronal migration, axon outgrowth, and axon guidance [21]. Recently, our group and others have also detected NOVA2 expression in the ECs of different normal human tissues, where it controls important steps of angiogenesis and vascular morphogenesis [22,23,24,25,26,27]. NOVA2 depletion, or its genetic knockout, in zebrafish prevents proper vascular development and differentiation [22,24]. Interestingly, NOVA2 knockdown in ECs allowed the identification of novel NOVA2-mediated changes in genes with relevant functions in vascular biology and angiogenesis [22,28,29]. Recently, NOVA2 has been reported significantly upregulated in tumor ECs of several cancer types, including ovarian, colorectal, hepatocellular, and head-neck squamous cell cancers. Negligible NOVA2 expression is found in the other cell types within the tumor [27,29,30]. Remarkably, high NOVA2 expression levels correlate with shorter overall survival in ovarian and colorectal cancer patients, and with shorter metastasis and relapse-free survival in colon cancer patients [27,29]. Interestingly, dysregulated NOVA2-mediated AS profiles are associated with the level of tumor vascularization or angiogenesis [27,29]. In addition, NOVA2 expression is induced in ECs under hypoxia, whereas vascular NOVA2 levels have been found to parallel HIF1α levels in colorectal cancer patients [30], uncovering hypoxia as a potential NOVA2 regulatory factor in cancer vasculature. While NOVA2 expression as a possible driver of tumor vascular aberrancies correlated with cancer metastasis and treatment resistance [31] is established, the specific AS events regulated by increased NOVA2 expression levels in ECs have not been identified yet.

## 2. Results

### 2.1. NOVA2 Overexpression Affects Several Splicing Switches in Angiogenesis Regulators 

**Figure 1:**
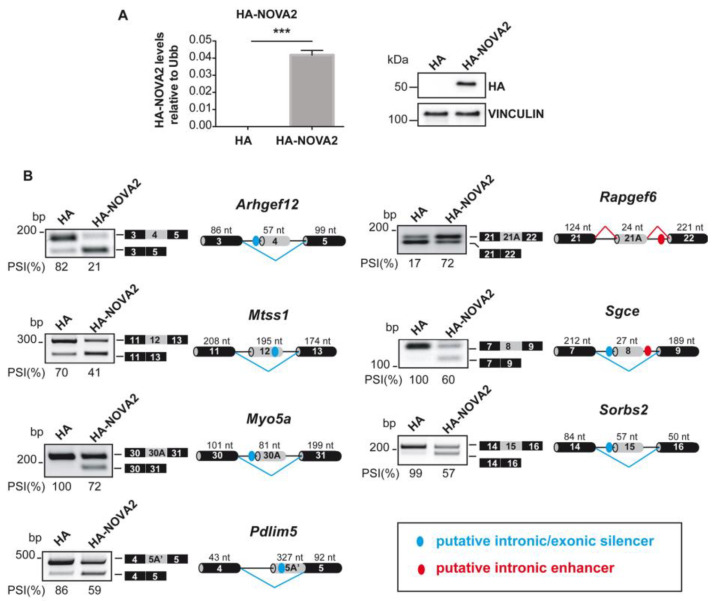
Novel AS events identified upon NOVA2 upregulation in mouse ECs. (**A**) Left panel: *HA-tagged NOVA2* mRNA levels in empty vector control (HA) or NOVA2 (HA-NOVA2)-overexpressing mouse ECs (moEC). Data represent the mean ± SEM (*n* = 3 independent experiments) *** *p* ≤ 0.001. Unpaired Student’s *t*-test. Right panel: Immunoblotting using anti-HA antibody in HA and HA-NOVA2 moEC. Vinculin is used as loading control. (**B**) RT-PCR analysis of selected NOVA2 splicing targets in moEC-overexpressing HA-tagged NOVA2. Transcripts generated from skipping/inclusion of the AS exon are represented near the corresponding RT-PCR bands. The percentages of exon inclusion (PSI) are also indicated. For each AS event, the genomic region containing the AS exon and the flanking sequences are represented; grey boxes: AS exons; black boxes: constitutive exons; blue/red dots: YCAY clusters predicted to function as NOVA silencer/enhancer; blue/red bars: NOVA-silenced/enhanced exon inclusion events.

To unveil the molecular pathways affected by NOVA2 upregulation, RNA-seq analysis was performed to compare the transcriptome of mouse ECs overexpressing NOVA2 and control mouse ECs (empty vector ECs) (Figure 1A). We identified 571 NOVA2-regulated AS events, in which the “cassette exon” modality—meaning that an alternative exon may be retained or skipped from the mature mRNA—was the most represented mechanism (291/571; 51%) (Table S1, sheet A and Figure S1A,B). By comparing these results with the AS events affected in NOVA2-depleted versus control ECs [29], we found a significant overlap (*p* value = 7.6 × 10^−5^ and again, most events reciprocally regulated were cassette exons (Figure S1C, Table S1, sheet B), thus expanding the list of NOVA2-regulated AS events in ECs [22,29].

Next, we performed a gene ontology (GO) analysis to identify which GO terms were significantly enriched in the AS events modulated by NOVA2 overexpression in ECs by using the MSigDB (https://www.gsea-msigdb.org/gsea/msigdb/; accessed on 19 April 2023; Mouse MsigDB v2023.1; San Diego, California) [32,33]. Consistent with the phenotype observed in NOVA2-depleted ECs [22], GO analysis found a significant enrichment for genes involved in cytoskeleton dynamics, cell adhesion, cell polarity, motility, and GTPase function (Figure S1D and Table S2), activities that are fundamental in several steps of angiogenesis [34]. Interestingly, we also found additional GO terms related to DNA repair, metabolic processes, cell cycle, autophagy, and organelle assembly, thus suggesting that NOVA2 may have a pleiotropic role in the endothelium (Table S2).

Considering MSigDB pathway collection, we also found that AS events altered by NOVA2 overexpression in ECs are related to signaling cascades mediated by growth factors and their receptors, including the Epidermal Growth Factor Receptor (EGFR) (Table S2). Notably, EGF plays crucial roles in tumor angiogenesis thanks to its ability to generate a favorable environment for the development of cancer vasculature [35]. Accordingly, EGFR is also one target of anti-angiogenic or tumor vasculature normalization approaches [36].

Collectively, our RNA-seq data suggest the existence of an intricate NOVA2-mediated AS decision network, which is involved in modulating critical aspects of EC biology as well as in molecular pathways related to tumor angiogenesis and cancer progression.

To validate our RNA-seq results, we analyzed the AS profile of several newly identified NOVA2-regulated AS events selected for their involvement in EC biology in physiological or pathological conditions (Table 1). 

By performing RT-PCR of RNA extracted from NOVA2-overexpressing ECs compared to empty vector control ECs, we successfully validated the AS profile of all the selected transcripts (Figure 1B). 

**Figure 2:**
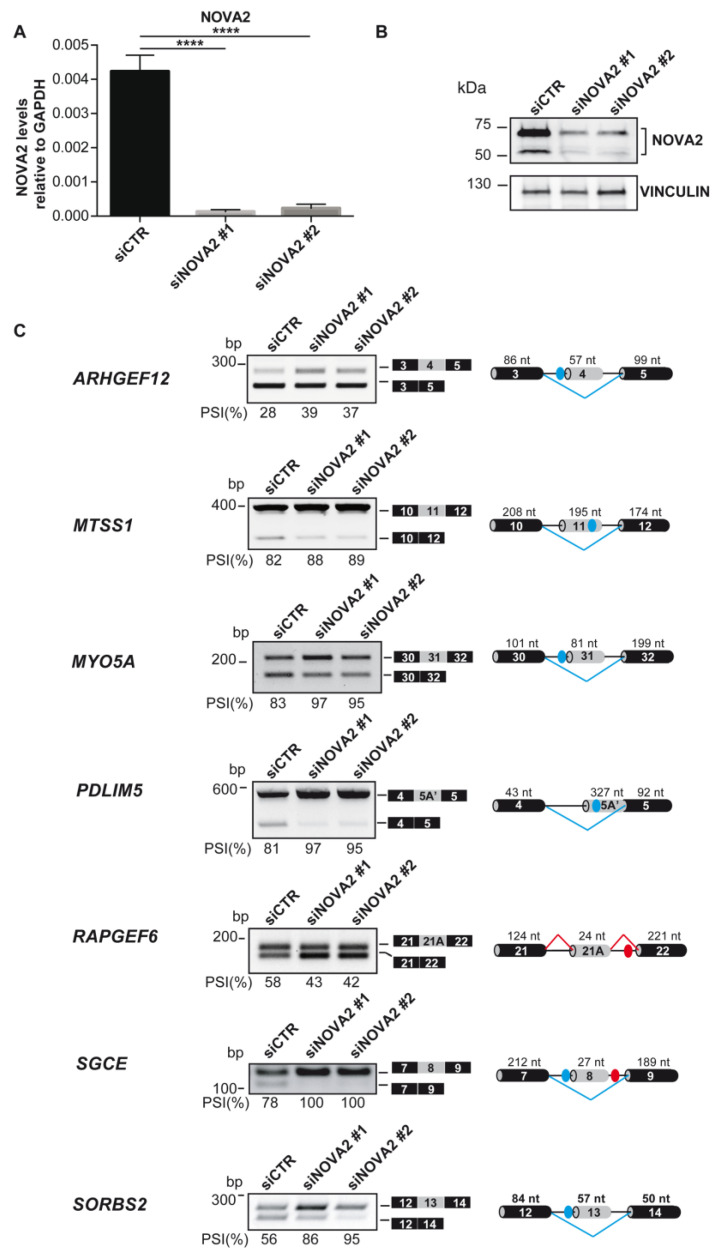
Validation of NOVA2-mediated AS events in human ECs knockdown for NOVA2. (**A**) *NOVA2* mRNA levels in HUVEC hTERT transfected with control (si*CTR*) or two different *NOVA2* siRNAs (si*NOVA2 #1*, si*NOVA2 #*2). Data represent the mean ± SEM (*n* = 3 independent experiments) **** *p* ≤ 0.0001. One-way ANOVA with multiple Tukey’s comparisons test. (**B**) NOVA2 immunoblotting in si*CTR,* si*NOVA2 #1* and si*NOVA2 #*2 HUVEC hTERT. (**C**) RT-PCR analysis of selected NOVA2 targets in si*CTR*, si*NOVA2 #1* and si*NOVA2 #*2 HUVEC hTERT. Transcripts generated from skipping/inclusion of the AS exon are represented near the corresponding RT-PCR bands. The percentages of exon inclusion (PSI) are also indicated. For each AS event, the genomic region containing the AS exon and the flanking regions are represented; grey boxes: AS exons; black boxes: constitutive exons; blue/red dots: YCAY clusters predicted to function as NOVA silencer/enhancer; blue/red bars: NOVA silenced/enhanced exon inclusion events.

Consistently, the AS of these transcripts was affected in the opposite direction by Nova2 depletion in ECs (Figure S2). By performing bioinformatic analysis with RBPmap (http://rbpmap.technion.ac.il accessed on 19 April 2023) [71] for all these genes, we identified putative NOVA2 binding sites (YCAY motifs) [72] located either in the AS exon or within the flanking intronic sequences (Figure 1B, Figures S2B and S3). In line with the possibility that NOVA2 directly regulates their AS patterns, the position of the putative NOVA2 binding sites and the type of AS event observed upon NOVA2 modulation were consistent with the known ability of NOVA2 to induce exon skipping when bound to exonic or upstream intronic YCAY motifs, while promoting exon inclusion when interacting with downstream clusters [72]. Importantly, the presence of putative NOVA2 binding sites and NOVA2-mediated AS regulation for all selected transcripts were conserved in human ECs (Figure 2 and Figure S4).

### 2.2. Vascular NOVA2 Expression Is Associated with Poor Prognosis in Gastric Cancer 

By using MSigDB and Enrichr (http://amp.pharm.mssm.edu/Enrichr/, accessed on 19 April 2023) [32,33,73,74], we searched for cancer terms uncovered by our newly identified NOVA2-mediated AS events (Table S1, sheet A). We found a statistically significant enrichment for terms related to gastric cancer (GC) and Helicobacter pylori, a crucial player for GC pathogenesis [75] (Table S3). Angiogenesis represents a key step in the progression of GC, and anti-angiogenic agents have been used for its treatment [76]. However, these strategies have shown modest therapeutic effects thus far [77,78,79]. Consequently, there is a growing interest in the discovery of new therapeutics targeting tumor vasculature in GC.

**Figure 3:**
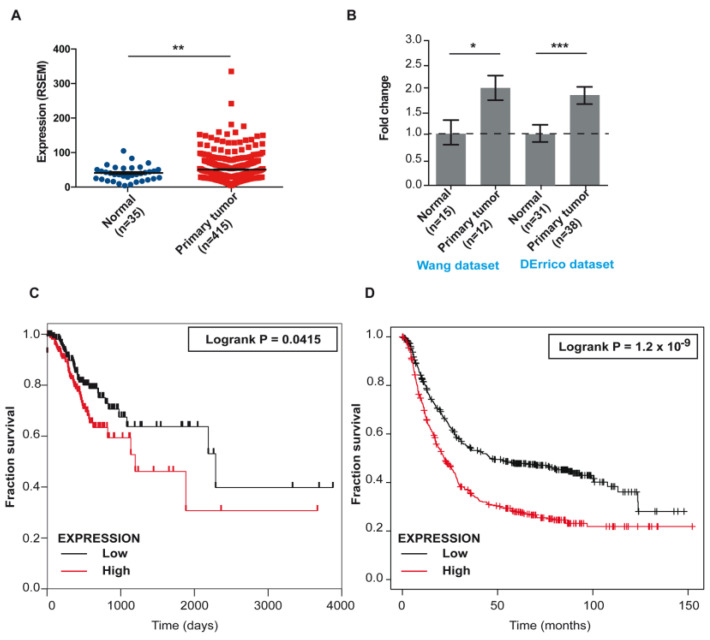
*NOVA2* expression levels are increased in GC and associated with poor overall patients’ survival. (**A**) Expression of *NOVA2* mRNA levels (mean ± SEM) in normal and GC samples from TCGA-STAD project (from TSVdb); Unpaired Student t-test with Welch’s correction (*p* = 0.0099). ** *p* ≤ 0.01. (**B**) Fold change of *NOVA2* expression in normal and GC samples from Wang dataset (GSE19826) and DErrico dataset (GSE13911) (probe: 206477_s_t, *p* = 0.0145 (* *p* ≤ 0.05) and *p* = 0.0003 (*** *p* < 0.001, respectively) with Unpaired Student’s *t*-test. (**C**) Kaplan–Meier plot of overall survival in GC patients from TCGA-STAD project classified according to *NOVA2* expression (cutoff: median) (red curve, high expression; black curve, low expression). Log-rank (Mantel–Cox) test (*p* = 0.0415). (**D**) Kaplan–Meier plot of overall survival in GC patients from GSE14210, GSE15459, GSE22377, GSE29272; GSE38749, GSE51105, GSE62254 datasets classified according to *NOVA2* expression (cutoff: median; probe: 206477_s_t; Log-rank (Mantel–Cox) test *p* = 1.2 × 10^−9^) (red curve, high expression; black curve, low expression).

Using *The Cancer Genome Atlas* (TCGA-STAD, Stomach Adenocarcinoma project) (http://gdac.broadinstitute.org accessed on 19 April 2023) [80] and Oncomine microarray GC datasets (www.oncomine.org accessed on 19 April 2023) [81], we found significantly elevated *NOVA2* expression in GC compared to the normal counterpart (Figure 3A,B and Table S4). Moreover, high *NOVA2* expression was significantly associated with poor overall survival in GC patients (Figure 3C,D). In the TCGA-STAD dataset, we found a statistically significant correlation between *NOVA2* expression, tumor size (according to the TNM classification of malignant tumors), and different GC histotypes (Figure S5A). In particular, high *NOVA2* expression is more frequently observed in the invasive diffuse-type adenocarcinomas (*p* = 0.0132) (Figure S5B). We did not find a significant difference in GC stages according to *NOVA2* expression considering all the different GC histotypes (Figure S5C). Stage I intestinal tumors are significantly (*p* = 0.0222) associated with low *NOVA2* expression levels (Figure S5D). Additionally, high *NOVA2* expression is more frequently observed in the most advanced grade 3 GC (chi-square 9.08; *p* = 0.0107) (Figure S5E). Notably, we also observed an enrichment of the microsatellite unstable (MSI) subtype in *NOVA2*-low-expressing GC samples (*p* < 0.0001). In contrast, an increased frequency of genomically stable (GS) subtypes correlates with high *NOVA2* levels (*p* < 0.0001) (Figure S5F). 

**Figure 4:**
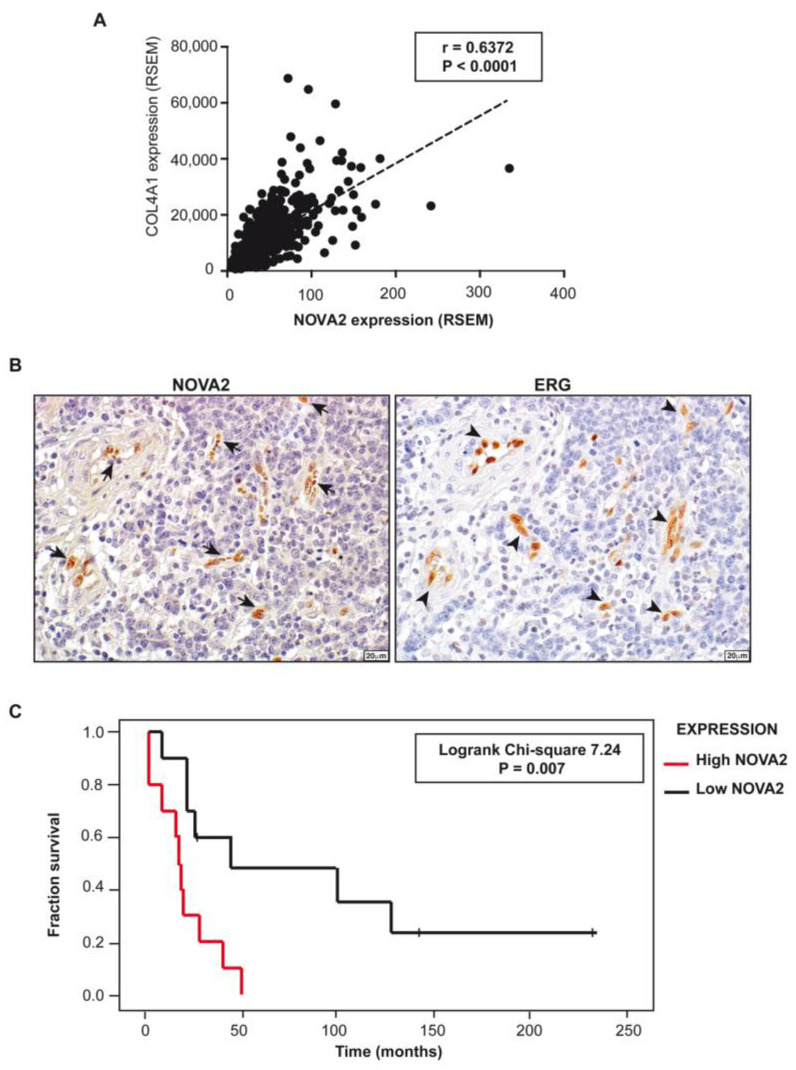
Expression of NOVA2 in gastric cancer vessels. (**A**) Correlation analysis showing a significant association between *COL4A1* and *NOVA2* expression levels from TGCA-STAD project (Pearson r = 0.6372, *p* < 0.0001). (**B**) Serial sections of GC samples (*n* = 27) stained for NOVA2 (left panel) or ERG (right panel). Arrows indicate NOVA2-positive nuclei of ECs. No immunoreactivity is present in the nuclei of tumor cells. Arrowheads indicate ERG-positive nuclei of ECs in the same area on a consecutive section of tissue. Scale bar: 20 μm. (**C**) Kaplan–Meier plot of overall survival in our cohort of GC patients classified according to *NOVA2* expression (red curve, high expression; black curve, low expression).

We found that *NOVA2* expression is significantly correlated to that of *Collagen type IV Alpha 1 Chain* (*COL4A1*)*,* a poor prognosis angiogenic marker for GC (Figure 4A) [82,83], raising the possibility that NOVA2 was selectively overexpressed in tumor ECs. To test this, we performed NOVA2 immunohistochemistry (IHC) analysis in a cohort of 27 GC patients. Intriguingly, NOVA2 expression was restricted to the nuclei of ECs (Figure 4B). Linear regression analysis showed a statistically significant correlation between the expression levels of NOVA2 and ETS-related gene (ERG) (R^2^ = 0.5845, *p* < 0.001), a specific EC marker [84]. 

The vessel-restricted expression of NOVA2 was confirmed in an independent cohort of GC patients (Figure S6A–D). Importantly, the percentage of NOVA2-positive ECs on the total of ECs stained with CD31 marker was higher in tumor sections compared with the adjacent normal tissues (Figure S6D and Table S5). At the protein level, high NOVA2 expression showed a trend in association with the presence of lymph node metastases (chi-square 3.28; *p* = 0.07). The association becomes statistically significant considering only the intestinal-type GCs (chi-square 5.13; *p* = 0.024). High NOVA2 expression was present in 70% of node-positive cases, compared with 14% of node-negative ones (chi-square 4.23; *p* < 0.05). In diffuse-type GCs, all cases showed the presence of lymph node metastases, and high NOVA2 was observed in 55.6% of cases (Table 2). In addition, high NOVA2 expression was more frequent in disease stage III (61%) than in stages I and II (33%) and in mismatch repair (MMR)-proficient cases (57%) than in MMR-defective cases (33%). 

With a follow-up of 20 years, in addition to lymph node metastases and diffuse histotype (not shown), high NOVA2 expression was found significantly associated with cancer-related death (log-rank chi-square 7.24; *p* = 0.007; Figure 4C). Both diffuse histotype and high NOVA2 expression were also proven to be unfavorable independent prognostic factors in multivariate Cox regression analysis (Table 3).

### 2.3. Alternative Splicing of RapGEF6 in Gastric Cancer Patients 

Based on our novel findings in GC, we validated the clinical relevance of the NOVA2-mediated AS in this cancer type. Among the novel NOVA2 targets discovered following its overexpression in ECs (Figure 1B), *RapGEF6* (also known as *PDZ-GEF2*) was identified. *RapGEF6* is highly expressed in retinal ECs [85] and plays crucial functions in ECs [61]. RapGEF6 is a member of the guanine nucleotide exchange factor (GEF) family, able to activate small GTPases, including the angiogenesis regulator Rap1 [65,86].

**Figure 5:**
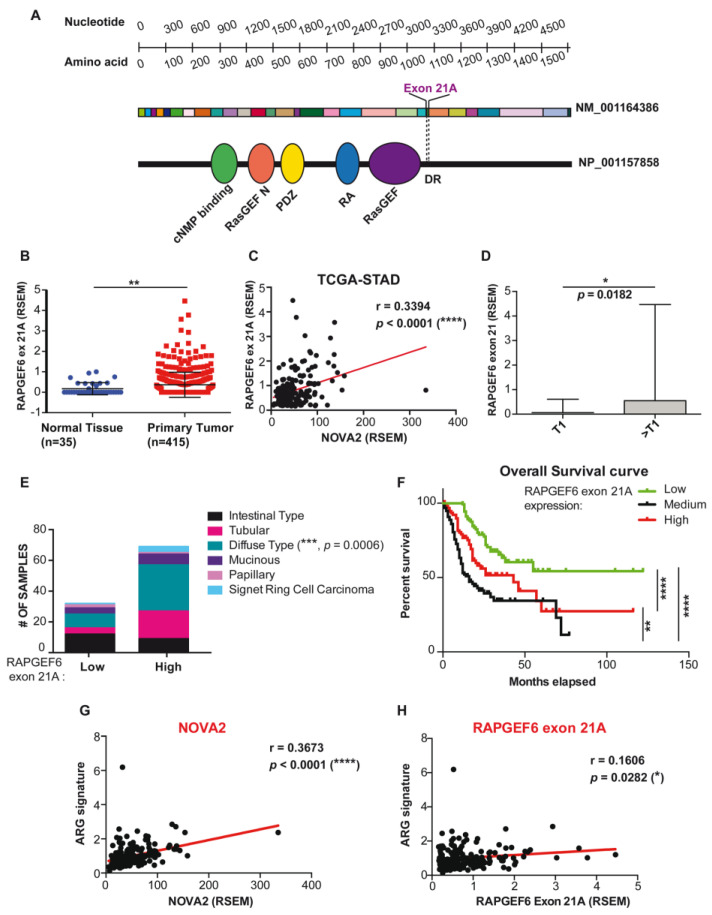
Expression of *RapGEF6* exon 21A in the TCGA-STAD dataset. (**A**) Coding transcript and protein domains of RapGEF6 adapted from DoChaP web tool. Black lines with numbers show the position in the transcript (nucleotides) and protein (amino acids). Different exons are represented in different colors; exon 21A is highlighted. RapGEF6 protein (NP_001157858) is depicted as a black line, with elliptical shapes representing functional domains: cyclic-nucleotide-binding domain (green); N-terminal domain for RasGEF-like protein domain (orange); post-synaptic density protein, disc large tumor suppressor, zonula occludens-1 protein (PDZ) domain (yellow); ras-associating domain (blue); ras-like guanine nucleotide exchange factor domain (purple). DR stands for disordered region encoded by exon 21A. (**B**) *RapGEF6* exon 21A in healthy donor (blue) and tumor patient (red) samples of TCGA-STAD dataset. *p* value was calculated with unpaired student *t*-test with Welch’s correlation. (**C**) Correlation between *NOVA2* and *RapGEF6* exon21A expression in TCGA-STAD samples. Linear regression (red line) and Pearson r coefficient with two-tailed *p* value are reported. (**D**) *RapGEF6* exon 21A expression in RSEM stratified as upper and lower quartiles according to tumor size (T1 and >T1). Unpaired student t-test. (**E**) Histotype distribution of STAD-GC tumors for low and high *RapGEF6* exon 21A expression (comparing lower and upper quartile). Fisher’s exact test. (**F**) Kaplan–Meier analysis of 10-year overall survival for STAD patients stratified for *RapGEF6* exon 21A expression in high (upper quartile, red line), medium (medium quartile, black line) and low (lower quartile, green line) levels. Log-rank (Mantel–Cox) test. *p* value legend: * *p*  <  0.05; ** *p* < 0.01; *** *p* < 0.001; **** *p*  <  0.0001. (**G**,**H**) *NOVA2* and *RapGEF6* exon 21A correlation with a gastric angiogenesis-related gene (ARG) signature.

We found that NOVA2 promotes the inclusion of exon 21A (of 24 nt) in the *RapGEF6* mRNA, both in mouse and human ECs (Figure 1B,C). By using the PFAM database (https://pfam.xfam.org/ accessed on 19 April 2023) [87] and the DoChaP web server (https://dochap.bgu.ac.il/ accessed on 19 April 2023) [88], we found that *RapGEF6* exon 21A encodes for a disordered region adjacent to the RasGEF domain (Figure 5A). Interestingly, it has been reported that exon 21A is important for the establishment of tissue-dependent protein–protein interaction networks [64]. Notably, exon 21A inclusion in the mature mRNA generates a RapGEF6 protein isoform with a reduced ability to interact with Rap1 [64], raising the possibility that NOVA2-mediated AS of *RapGEF6* could be responsible for differential Rap1 activity regulation in a spatial- or temporal-specific manner.

Given the importance of *RapGEF6* exon 21A splicing regulation in EC biology, we further investigated *RapGEF6* exon 21A expression levels in the TCGA-STAD dataset.

Using TSVdb, a web tool allowing for the comparison of isoform expression among clinical subgroups (http://tsvdb.com/index.html accessed on 19 April 2023) [89], we found that *RapGEF6* exon 21A is upregulated in TCGA-STAD tumor samples compared with normal ones (Figure 5B and Table S6). Moreover, a positive and statistically significant correlation was observed between *NOVA2* and *RapGEF6* exon 21A expression levels in primary tumor samples (Figure 5C and Table S6). Tumors with higher size/extension (T2, T3, T4 according to the TNM classification of malignant tumors) show higher levels of *RapGEF6* exon 21A compared with smaller ones (T1, T1a, T1b) (Figure 5D and Table S5). Furthermore, *RapGEF6* exon 21A expression recapitulates *NOVA2* in the TCGA-STAD histotype. *RapGEF6* exon 21A was more expressed in invasive diffuse-type adenocarcinomas, where *NOVA2* levels were higher compared with other histotypes (Figure 5E and Table S6). Importantly, cancer patients with high *RapGEF6* exon 21A expression show shorter overall survival compared with patients displaying low or medium *RapGEF6* exon 21A expression (Figure 5F and Table S6). In addition, as with NOVA2 (Figure 5G and Table S6), a consistent positive correlation was found between *RapGEF6* exon 21A expression and an established GC angiogenesis signature (Figure 5H and Table S6). 

## 3. Discussion

Gastric cancer (GC), the third leading cause of cancer-related deaths worldwide [90], is strongly dependent on angiogenesis. Anti-angiogenic therapies are currently used for GC treatment in clinical settings [77]. We found that the AS factor NOVA2 is overexpressed in ECs of GC with a significant prognostic value. Indeed, high NOVA2 levels correlate with poor overall survival of GC patients and with the presence of lymph node metastases. Aberrant NOVA2 overexpression could lead to splicing errors generating protein variants involved in the development of the cancer vasculature. To identify AS changes mediated by increased NOVA2 expression in the endothelium, we compared the transcriptome of ECs stably overexpressing NOVA2 versus control ECs. This allowed us to comprehensively identify novel AS events promoted by NOVA2 upregulation. The novel NOVA2-mediated AS changes we identified not only expand the list of previously known NOVA2 targets, but also uncover further functional implications of NOVA2 in ECs. Indeed, in addition to transcripts encoding for factors involved in apical–basal polarity, actin polymerization dynamics and cytoskeletal remodeling, we found that NOVA2 upregulation alters the AS profile of transcripts encoding for factors related to DNA repair, metabolic processes, the cell cycle, autophagy, and organelle assembly. Moreover, enrichment pathway analysis of the AS events altered by NOVA2 overexpression in ECs also showed possible NOVA2 involvement in signaling cascades mediated by growth factors and their receptors, including the EGFR (Table S3). EGFR is overexpressed in a significant fraction (27%–64%) of gastric tumors [91,92], where its oncogenic role has been well-characterized [93]. Notably, EGFR inhibition has been tested in clinical studies [93] and evaluated to enhance the efficacy of anti-cancer drugs by normalizing the tumor vasculature [36]. However, our results suggest that anti-cancer strategies targeting EGFR signaling in cancer vasculature should take into account the expression of NOVA2-mediated AS isoforms in tumor ECs as potential mechanisms of resistance.

Based on the essential role played by small GTPases (and their regulators) during angiogenesis, including the maintenance of endothelial integrity, as well as in cancer progression and metastasis [86,94,95], we focused our attention on the newly identified NOVA2 target *RapGEF6. RapGEF6* encodes for a member of the guanine nucleotide exchange factor (GEF) family. *RapGEF6* is highly expressed in the retinal ECs [85], whereas its depletion affects the organization of the cell–cell contacts, with the formation of irregular membrane invaginations [61].

RapGEF6 is an upstream activator of Rap1, which, similarly to NOVA2, modulates the organization of adherens junctions, the acquisition of cell polarity, and the formation of a correct vascular lumen [86,96]. Rap1-defective signaling is associated with cerebral cavernous malformations (CCMs), which are vascular abnormalities within the CNS characterized by the presence of defective small blood vessels with thin walls [86,96]. This type of morphology, with a severely altered lumen, is somehow reminiscent of the morphology of the tumor vasculature in which NOVA2 expression is increased. 

Interestingly, constitutive activation of Rap1 is also involved in cancer progression, while its inhibition counteracts carcinogenesis, metastasis, and chemoresistance [97].

We found that high NOVA2 promotes the inclusion of *RapGEF6* exon 21A in the mature mRNA, generating a protein variant previously shown to have a reduced ability to interact with Rap1 [64]. This alternatively spliced exon encodes for a disordered region adjacent to the RasGEF domain of the RapGEF6 protein.

Tissue-specific AS exons frequently encode for sequences predicted to be highly disordered [64]. In addition, proteins containing tissue-regulated AS exons that overlap with intrinsically disordered regions (IDRs) are involved in significantly more protein–protein interaction networks compared with other proteins [64]. Our findings raise the possibility that the NOVA2-mediated AS regulation of *RapGEF6* could promote or disrupt partner interactions, thus leading to differential modulation of Rap1 activity in a spatial- or temporal-specific manner.

We also found that in GC patients there is a positive and statistically significant correlation between the AS pattern of *RapGEF6* exon 21A and *NOVA2* expression levels. As with NOVA2, high *RapGEF6* exon 21A expression is associated with a previously described angiogenic signature and reduced overall survival of GC patients. Collectively, our data are in line with the possibility that the NOVA2/RapGEF6 circuit in tumor ECs contributes to the phenotypical and functional vascular aberrancies observed in GC patients. However, the elucidation of the NOVA2/RapGEF6 circuit’s functional role in tumor vasculature requires further studies. Nevertheless, our findings contribute to improving the current understanding of GC biology and to highlight the importance of a poorly understood aspect of gene expression regulation (such as AS) in the context of GC vasculature, offering opportunities for the discovery of novel biomarkers and therapeutic targets.

## 4. Materials and Methods

### 4.1. Immunohistochemistry

Twenty-seven cases of formalin-fixed paraffin-embedded (FFPE) from GC patients who underwent surgery between 1986 and 1987 were retrieved at the Surgical Pathology Unit of the “Circolo Hospital and Macchi Foundation” in Varese (Italy). The cases are part of a well-characterized series of advanced GCs with clinical information and long-term follow-up. Cases were selected on the basis of the tumor type and the available materials. None of the patients underwent either neoadjuvant or adjuvant chemotherapy. Clinical data are summarized in Table 2.

Formalin-fixed paraffin-embedded 3 μm sections were cut consecutively. One section was immunostained for NOVA2 (goat anti-NOVA2, *C-16* Santa Cruz Biotechnology, Dallas, TX, USA, Cat# sc-10546, RRID:AB_2151558; 1:100), whereas the consecutive section was stained with an anti-ERG antibody (rabbit anti-ERG, *EPR3864* Ventana Medical Systems, Oro Valley, AZ, USA, Cat# 790-457; undiluted). Sections were deparaffinized and rehydrated. Blocking of endogenous peroxidase was obtained by topping sections with 3% hydrogen peroxide in distilled water for 20 min. Antigen retrieval was performed using citrate buffer solution (10 mM pH 6) in a microwave oven for 20 min, both for NOVA2 and ERG antigens. Sections were incubated overnight (ON) with primary antibodies in a refrigerator. They were further incubated with biotinylated rabbit anti-goat IgG (H + L); (Vector Laboratories, Newark, CA, USA, Cat# BA-5000) and ABC peroxidase (Vectastain^®^ Elite ABC-HRP kit, Vector Laboratories, Cat# PK-6100) for anti-NOVA2 antibody and with MACH 4^TM^ HRP polymer (Biocare Medical, Pacheco, CA, Cat# M4U534) for anti-ERG antibody, respectively. Reactions were developed using DAB (3,3-diaminobenzidine tetrahydrochloride, Cat#D5905, Sigma Life Science, Burlington, MA, USA) and nuclei were counterstained with hematoxylin.

NOVA2- and ERG-positive cells were counted along the invasive margin in 5 consecutive high-power fields (HPFs; 400X area; 0.19 mm^2^ per field). For each antibody, the median value of positive cells was used as a cutoff to stratify cases into low (≤33 cells/HPF for NOVA2 and ≤35 cells/HPF for ERG) or high expression. Statistical analysis of IHC raw data was performed with SPSS Statistics Software, Chicago, IL, USA (version 23).

In addition, 4 µm tissue sections were obtained from paraffin tissue blocks from gastric cancers retrieved from the archive of Pathology of the U.O. Anatomia Patologica (Spedali Civili of Brescia, Italy). NOVA2 IHC detection was obtained by using anti-NOVA2 (polyclonal rabbit, 1:100; Atlas Antibodies, Cat# HPA045607, RRID: AB_10962628) antibody. Antigen retrieval was performed using EDTA Buffer (pH 8.0), whereas signal detection was obtained using the Novolink Polymer System (Novocastra, Leica Biosystems, Milan, Italy) followed by DAB. Stained slides were acquired using the Aperio CS2 digital scanner and ScanScope software (v. 10.1, Leica Biosystems, Vista, CA, USA, Cat#12.03.5048;). Images were analyzed using ImageScope software (v. 12.4.6, Leica Biosystems, Milan, Italy). The region of interest consisted of the tumor area and normal gastric tissue; necrotic areas were excluded from the selection. The IHC Nuclear Image Analysis algorithm (Leica biosystems) was set up for the analysis to identify categories of strong and weak positive cells based on the signal intensity as described in [27]. Data are expressed as the absolute number of NOVA2-positive cells per mm^2^ and as fractions of strong and weak positive cells. Double IHC for NOVA2 and CD31 (clone PECAM-1, 1:100; Leica Biosystems, Cat# CD31-607-L-CE, RRID:AB_2935723) was performed as previously described in [27]. Data are expressed as the number of NOVA2-positive cells on the total of CD31 positive cells.

### 4.2. Cell Cultures

Mouse endothelial cells (moEC) [29] stably overexpressing human NOVA2 or knockdown for *Nova2* (as well as their relative controls) were generated by lentiviral transduction of pLenti-GIII-CMV-HA-NOVA2 and control HA (THP Medical Products, Wien, Austria) or GIPZ Lentiviral Nova2 short-hairpin RNA (shRNA) for NOVA2 and control shRNA (Open Biosystems) as described in [22]. MoEC overexpressing or knockdown for *Nova2* were cultured in DMEM-high glucose with 1mM sodium pyruvate (Euroclone, Milan, Italy, Cat# ECB7501L) supplemented with 10% FBS (Euroclone, Cat# ECS500L), 2 mM L-glutamine (Euroclone, Cat# ECB3000D), 100 U/L penicillin/streptomycin (Euroclone, Cat# ECB3001D), 25 mM HEPES (Euroclone, Cat# ECM0180), 100 g/mL heparin (from porcine intestinal mucosa, Merck Millipore, Saint Louis, MA, USA, Cat# H3149), 5 μg/mL EC growth supplement (ECGS from bovine pituitary gland, Merck Millipore, Cat# E2759) under puromycin selection (3 μg/mL, InvivoGen, San Diego, CA, USA, Cat# ant-pr-1). Since NOVA2 expression is regulated by EC density [22], for the splicing analysis of NOVA2 targets moEC overexpressing HA-tagged NOVA2 were grown as sparse (500,000 cells in 100 mm Petri dishes), while NOVA2-knockdown moEC were seeded as confluent monolayers (500,000 cells in 35 mm Petri dishes).

HUVEC/TERT2 (Evercyte, Wien, Austria, Cat# CHT-006-0008) were cultured in EBM™ Basal Medium (Lonza, Basel, Switzerland, Cat# CC-3156) with selected supplements from EGM™ SingleQuots™ Kit (Lonza, Cat# CC-4133), namely BBE (bovine brain extract), hEGF (human epidermal growth factor), hydrocortisone solution and ascorbic acid solution, plus 10% FBS (Euroclone, Cat# ECS500L) and 20 μg/mL G418 (Gibco, Dublin, Ireland, Cat# 10131035). 

All cells were routinely tested for the absence of mycoplasma by qPCR-based service (Micoplasmacheck Barcode, eurofins Genomics, Ebersberg, Germany).

### 4.3. RNA Interference

To knock down *NOVA2* expression in HUVEC/TERT2, we used two different siRNA oligos from Sigma-Aldrich (Merck Millipore, siNOVA2 #1 MISSION siRNA ID SASI_HS01_00220812 and siNOVA2 #2 MISSION siRNA ID SASI_HS01_00220812) and the corresponding negative control (Merck Millipore, MISSION siRNA Universal Negative Control #2). Transfection was performed with Lipofectamine RNAiMax (ThermoFisher Scientific, Waltham, MA, USA, Cat# 13778030) following the manufacturer’s instructions. Two subsequent transfections (with 24 h intervals) were performed with 30 nM siRNA in Opti-MEM (Gibco, Cat# 31985047) for 5 h, then replaced with complete EGM. Cells were collected for RNA/protein analysis 48 h after the second transfection. 

### 4.4. Western Blot Analysis

Total proteins were extracted by using Laemmli buffer (4% SDS, 16% glycerol, 40 mM Tris-HCl pH 6.8), and cell lysates were quantified with a Pierce BCA protein assay kit (Thermo Fisher Scientific, Cat #23225). Proteins (20 μg/lane) were resolved by SDS-PAGE on precast gels 4–15% (Biorad, Hercules, CA, USA, Cat #4568084) and transferred to nitrocellulose membranes with Trans-Blot Turbo (Biorad). Proteins of interest were visualized using specific antibodies, followed by peroxidase-conjugated secondary antibodies. The following primary antibodies were used: goat anti-NOVA2 C-16 (1:200, Santa Cruz Biotechnology, Cat# sc-10546, RRID: AB_2151558), mouse anti-Vinculin (1:10,000, Cell Signaling, Cat# MAB3574, RRID:AB_2304338), rat anti-HA High Affinity (1:1000, Roche, Cat# 11867423001, RRID:AB_390918). Secondary antibodies were purchased from Jackson ImmunoResearch (Cambridgeshire, United Kingdom): anti-Mouse (1:5000, Cat#105-035-146), anti-Goat (1:5000, Cat#705-035-147) anti-Rat (1:5000, Cat# 112-035-175). Chemiluminescent signal was detected by using ECL LiteAblot Plus/Extended/Turbo (Euroclone, Cat#EMP011005/#EMP013001/#EMP013001) and acquired by ImageQuant LAS 500 chemiluminescence CCD-camera (GE Healthcare, Chicago, IL, USA).

### 4.5. RNA-seq and Splicing Analysis

RNA-seq libraries were prepared from total RNA of two duplicates of control and NOVA2-overexpressing ECs and sequenced on an Illumina HiSeq2500 (minimum of 71.8 million reads, 100-nucleotide (nt) paired-end reads for each run). Reads were aligned with a mouse C57BL/6J reference genome mm9 assembly using *vast-tools* [98]), providing “corrected” reads per kilobase per million mapped reads (cRPKMs) [99] and raw counts.

To identify and quantify all major types of AS events affected by NOVA2 upregulation, we used *vast-tools* [98], a program that maps RNA-seq reads to wide-ranging sets of annotated and novel splice junctions and generates confident estimates of the percentage of alternative exon inclusion in a given sample (PSIs, ‘Percent Spliced In’, for exons; PIR, ‘Percent Intron Retention’, for introns). Data were analyzed considering the reads of duplicates of NOVA2-overexpressing ECs compared with control cells. Reads in EC knockdown for NOVA2 were also considered [22]. A minimum dPSI > 5 between each replicate in the overexpression and in the knockdown experiment was imposed, then data were filtered to identify differentially spliced genes specifically in NOVA2-overexpressing ECs as those with absolute dPSI ≥ 10.

To identify overlapping splicing events between NOVA2-gain and loss-of-function ECs, we compared the list of novel NOVA2 targets upon NOVA2 overexpression identified in this study (Table S1) with previously published data reporting NOVA2-mediated AS events in NOVA2-knockdown ECs [22,29]. We used the *GeneOverlap* R package to statistically define the overlap of splicing events. Fisher’s exact test was used to assess the statistical significance of the overlapping. 

### 4.6. Functional Enrichment Analysis

The gene ontology (GO) and the pathway-enrichment analyses were performed using the MSigDB web tool (https://www.gsea-msigdb.org/gsea/msigdb/, accessed on 19 April 2023; Mouse MsigDB v2023.1) [32,33]. The Enrichr web tool (http://amp.pharm.mssm.edu/Enrichr/, accessed on 19 April 2023) [73,74] was used to interrogate the Orphanet Augmented 2021 database. Entries were ranked accordingly to their adjusted *p* values.

### 4.7. Binding Site Prediction for NOVA2 Protein on Pre-mRNA of Target Genes

Prediction of NOVA2 binding sites was carried out with RBPmap (https://rbpmap.technion.ac.il/, accessed on 22 February 2022) computational tool. For human and mouse genes, NOVA2 binding sites were searched in an alternative exon sequence ±200 nt flanking the alternative splicing exon, and the initial 200 nt of the upstream intron and the last 200 nt of the downstream intron. We selected default stringency parameters and NOVA1 motifs (*aucac* and *uucauaa*). We were forced to use NOVA1 motifs since NOVA2 is not listed by the tool, considering that NOVA2 and NOVA1 have been found to associate with identical sequences [100].

### 4.8. RNA Extraction, RT–PCR, and RT–qPCR

Total RNA was isolated using the RNeasy Mini Kit (QIAGEN, Germantown, MD, USA, Cat# 74106) according to the manufacturer’s instructions and then treated with DNase (Ambion, Naugatuck, CT, USA, Cat# AM1907). cDNAs were synthesized with a Superscript IV RT cDNA synthesis kit (Invitrogen by ThermoFisher Scientific, Cat# 18080051) using 500–1500 ng of total RNA. An aliquot of the RT reaction (1–2 μL) was then PCR-amplified (with GoTaq G2 Flexi DNA Polymerase, Promega, Madison, WI, USA, Cat# M7805). For quantitative PCR (qPCR), cDNA was amplified with QuantiTect SYBR Green PCR (QIAGEN, Cat# 204145) using the LightCycler 480 (Roche, Basel, Switzerland). Target transcript levels were normalized to those of reference genes. The expression of each gene was measured in at least three independent experiments. All PCR products were verified by sequencing. Primers are listed in Table S7. For PSI calculation (where PSI is defined as the percentage ratio of full-length transcripts over the sum of full-length and exon-skipped transcripts), the intensity of bands in agarose gel was assessed with Image J software (version 1.53e National Institute of Mental Health, USA).

### 4.9. TCGA-STAD Transcriptomic and Clinic-Pathological Analyses

TCGA-STAD cancer omics data and patient clinical information were retrieved from the cBioportal for Cancer Genomics (https://www.cbioportal.org, accessed on 19 April 2023) [101,102]. *NOVA2* and *RAPGEF6* exon 21A expression levels (RSEM) were downloaded from TSVdb (http://tsvdb.com/plot.html, accessed on 19 April 2023). GC patients were stratified with median cutoff according to *NOVA2* (low or high) expression levels. Tumor stage, pathological T category, and tumor grade (G) were defined according to the American joint committee on cancer code. Samples in which *NOVA2* levels or staging information were not present were excluded from the analysis.

### 4.10. Correlation Analyses

The correlation between *COL4A1* expression and *NOVA2* expression in the TCGA-STAD dataset was tested as Pearson’s correlation coefficient with GraphPad Prism 6. The association between protein expression and the discrete clinicopathological variables listed in Table 2 was tested with the Pearson chi-square test; the correlation of patients’ survival with *NOVA2* expression was estimated using the Kaplan–Meier limit method. Statistical differences were calculated using the Log-rank Test (SPSS Statistics Software^®^, version 23). The angiogenesis-related gene expression signature of gastric patients (ARG) from the TCGA-STAD dataset was constructed according to the expression levels of a series of angiogenesis-related genes predicting prognosis [103]: *ITGAV*, *FSTL1*, *LUM*, *POTN*, *VCAN*, *COL5A2*, *COL3A1*, *TIMP1*, *SPP1*, *OLR1*, *STC1*, *APOH*, *SLCO2A1*, *NRP1*, *POSTN*, *VTN*, *SERPINA5*, *LPL* and *KCNJ8*. ARG was calculated as the average of normalized gene expression level (relative to the mean of expression in the TCGA-STAD dataset) of each gene of the signature (Table S6). Correlation of *NOVA2* and *RAPGEF6* exon 21A expression with gastric ARG was assessed through Pearson’s correlation coefficient. 

### 4.11. Oncomine Database Analysis

Dataset transcriptomic data analyses of GC versus normal samples were retrieved in Oncomine database (http://www.oncomine.org accessed on 19 April 2023) [104]. *NOVA2* expression values were obtained by reporter probe 206477_s_t. The Wang Gastric dataset is comprised of 27 samples, 15 normal mucosa and 12 cancers [105], while the DErrico dataset consists of 38 GC and 31 normal samples [106]. The two-tailed unpaired Student’s t-test was used to assess significance.

### 4.12. Survival Analysis

For survival analysis of the STAD-TCGA cohort of patients, a Kaplan–Meier plot at 10 years was constructed, stratifying patients according to low and high *NOVA2* expression (cutoff: median). The log-rank Mantel–Cox test (GraphPad Prism) was used to determine statistical significance between the two defined groups.

The Kaplan–Meier Plotter web tool (https://kmplot.com/analysis/index.php?p=background, accessed on 19 April 2023) [107] was used for survival analysis of GC patients of all deposited GSE datasets cited in Figure 1D using a 206477_s_t NOVA2 probe and splitting patients by *NOVA2* expression median, maintaining all the other parameters as the default. For the overall survival curve of STAD patients at 10 years relative to *RapGEF6* exon 21A expression, patients were divided into three groups (low, high, and medium expression) according to quartiles, and the significance of the observed differences between the upper and lower quartiles was assessed with the log-rank Mantel–Cox test.

### 4.13. Statistic Reproducibility

When two groups were compared, paired or unpaired Student’s two-tailed *t*-tests were used to determine statistical significance. For contingency analyses, chi-square and Fisher’s exact tests were used to compare two or more groups, respectively, and to determine statistical significance (GraphPad Prism v. 6 and 9, San Diego, CA, USA).

## Figures and Tables

**Table 1:** Selected NOVA2-regulated AS exons involved in processes relevant for EC angiogenic behavior.

NOVA2 Targets in ECs	Function	NOVA2-Mediated AS Event
***ARHGEF12***	ARHGEF12 is an endothelial-enriched Rho-GEF (guanine nucleotide exchange factor) specifically activating the small GTPase Rho [37,38]. It interacts with plexin-B1, which mediates Semaphorin 4D-induced Rho signaling controlling angiogenesis [39]. ARHGEF12 mediates sphingosine-1 phosphate receptor 2 signaling, which inhibits EC sprouting through RhoC activation [40]. It acts downstream of ICAM-1 to increase RhoA-mediated cytoskeletal rearrangements in ECs [41]. Finally, it is involved in mechanical-force-induced activation of RhoA and cytoskeletal remodeling leading to EC reorientation [42].	NOVA2 regulates skipping of the exon 4 residing in a region, predicted as disordered by *disopred2*, contiguous to the PDZ domain involved in plexin-B1 binding [43].
***MTSS1***	*MTSS1* encodes for the metastasis protein suppressor protein 1, acting as a scaffold protein for multiple partners to regulate actin dynamics, formation of lamellipodia, membrane ruffles, filopodia-like structures, and disassembly of actin stress fibers [44,45,46,47]. MTSS1 promotes cell–cell junction formation and stability by activating the small GTPase Rac1 [48]. It mediates cell polarity and regulates the motility response to growth factors [49,50]	NOVA2 promotes skipping of the exon 12 that encodes for a predicted disorder region rich in Ser (putative phosphorylation site) located between the IMD (involved in actin bundling) and WH2 (involved in actin binding) domains.
***MYO5A***	MYO5A is an actin-based motor protein involved in cytoplasmic vesicle transport and anchorage, spindle-pole alignment, mRNA translocation and cell polarity [51]. In ECs, it regulates von Willebrand factor exocytosis [52]. MYO5A is involved in the early events of the formation of primary cilia [53], which are enriched in nascent blood vessels [54].	NOVA2 promotes skipping of the exon 30A (also known as exon D), which encodes for a region that is essential to interact with Rab10 and Rab8 proteins [55]. These proteins are important for the Golgi trafficking in epithelial cell polarization [56] and the biogenesis of Weibel–Palades granules containing von Willebrand factor [57].
***PDLIM5***	PDLIM5 is a cytoskeleton-associated scaffold protein regulating actin dynamics, cell architecture, cell migration, and gene transcription [58,59,60].	NOVA2 promotes skipping of exon 5A’, which encodes for a region neighboring actin-binding functional-domain PDZ.
***RAPGEF6***	RAPGEF6 is a guanine nucleotide exchange factor for the small GTPase Rap1. It plays a critical role in the maturation of adherens junctions and mechanoresponses of the Hippo pathway [61,62,63].	NOVA2 promotes the inclusion of exon 21A, encoding for a predicted disordered region downstream the RasGEF domain. This last domain is known to lower RAPGEF6 interaction with RAP1 [64], an important regulator of angiogenesis [65].
***SGCE***	*SGCE* encodes for sarcoglycan epsilon, part of the dystrophin-associated glycoprotein complex linking the cytoskeleton to the extracellular matrix. It may contribute to membrane stabilization and signal transduction in the cerebrovascular system and in lung ECs [66,67].	NOVA2 promotes skipping of exon 8, encoding for a region predicted to be disordered with a peculiar expression in the CNS [68].
***SORBS2***	SORBS2 is an adapter between ABL kinases and actin cytoskeleton. In ECs it plays a role in the maintenance of vascular lumens by balancing endothelial cytoskeletal dynamics and cell–matrix adhesion [69].	NOVA2 promotes skipping of exon 15, which encodes for portion residing in a large disordered region possibly affecting protein-protein interactions [70].

**Table 2:** Correlation between clinico–pathological data and expression of NOVA2 and ERG.

	No. of Cases	NOVA2*^high^* No. of Cases (%)	ERG*^high^* No. of Cases (%)
**No. of cases**	27	14 (51.9%)	14 (51.9%)
**Mean Age years (range)**	65 (33–85)	66 (51–85)	67 (52–85)
**Sex**			
Men	16	9 (56.3%)	9 (56.3%)
Women	11	5 (45.5%)	5 (45.5%)
**Histotype (Lauren classification)**			
Intestinal	17	8 (47.1%)	10 (58.8%)
Diffuse	9	5 (55.6%)	3 (33.3%)
Undifferentiated	1	1 (100%)	1 (100%)
**Cellular Grade**			
G2	7	4 (57.1%)	2 (28.6%)
G3	20	10 (50.0%)	12 (60.0%)
**pT**			
Intestinal	1	1 (100%)	1 (100%)
Diffuse	14	7 (50%)	8 (57.1%)
Undifferentiated	12	6 (50.0%)	5 (41.7%)
**pN**			
pN0	8	2 (25%) *	4 (50%)
>pN0	19	12 (63.2%) *	10 (52.6%)
**STAGE**			
I	1	1	1
II	8	2 (25%)	4 (50%)
III	18	11 (61.1%)	9 (50%)
**Survival** ^§^			
Cancer-related deaths	17	10 (58,8%) *	7 (41.2%)
Alive/deaths by other causes	9	3 (33.3%) *	6 (66.7%)
**Mismatch Repair System**			
MMR-Defective (MMR-D)	6	2 (33,3%)	4 (66.7%)
MMR-Proficient (MMR-P)	21	12 (57.1 %)	10 (47.6%)
**Follow-up: Mean months (range)**	93.4 (1–272)	69.8 (1–264)	105.2 (1–264)

Legend: MMR mismatch repair system. **^§^** Data not available for all cases. * Presence of a trend towards statistically significant association.

**Table 3:** Multivariate analysis for cancer-related death with follow-up to 240 months (Cox Regression).

Clinical Variable	*p* Value	OR	95% CI
High NOVA2	0.005	5.2	1.6–16.7
Diffuse GCs	0.014	4.3	1.3–1.4

## Data Availability

Genes and transcripts data, patient survival, and clinical information that support the findings of this study are available through cBioportal for Cancer Genomics (https://www.cbioportal.org accessed on 19 April 2023). Transcriptomic and clinical data used in this study have been included in Tables S4 and S6. RNA-seq data are openly available in the GEO (Gene expression omnibus) database, under the accession number GSE224823.

## Supplementary Materials

The following supporting information can be downloaded at: https://www.mdpi.com/article/10.3390/ijms24098102/s1.
