# Supplementary material for: Alternative Splicing Changes Promoted by NOVA2 Upregulation in Endothelial Cells and Relevance for Gastric Cancer
Source: Int J Mol Sci. 2023 Apr 30;24(9):8102. doi: 10.3390/ijms24098102 (PMC10178952; doi:10.3390/ijms24098102)
Supplement: Supplementary file 1 [file ijms-24-08102-s001.zip › ijms-2343297-supplementary.pdf]

International Journal of *Molecular Sciences*

MDPI

Supplementary materials

Alternative splicing changes promoted by NOVA2 upregulation in endothelial cells and relevance for gastric cancer

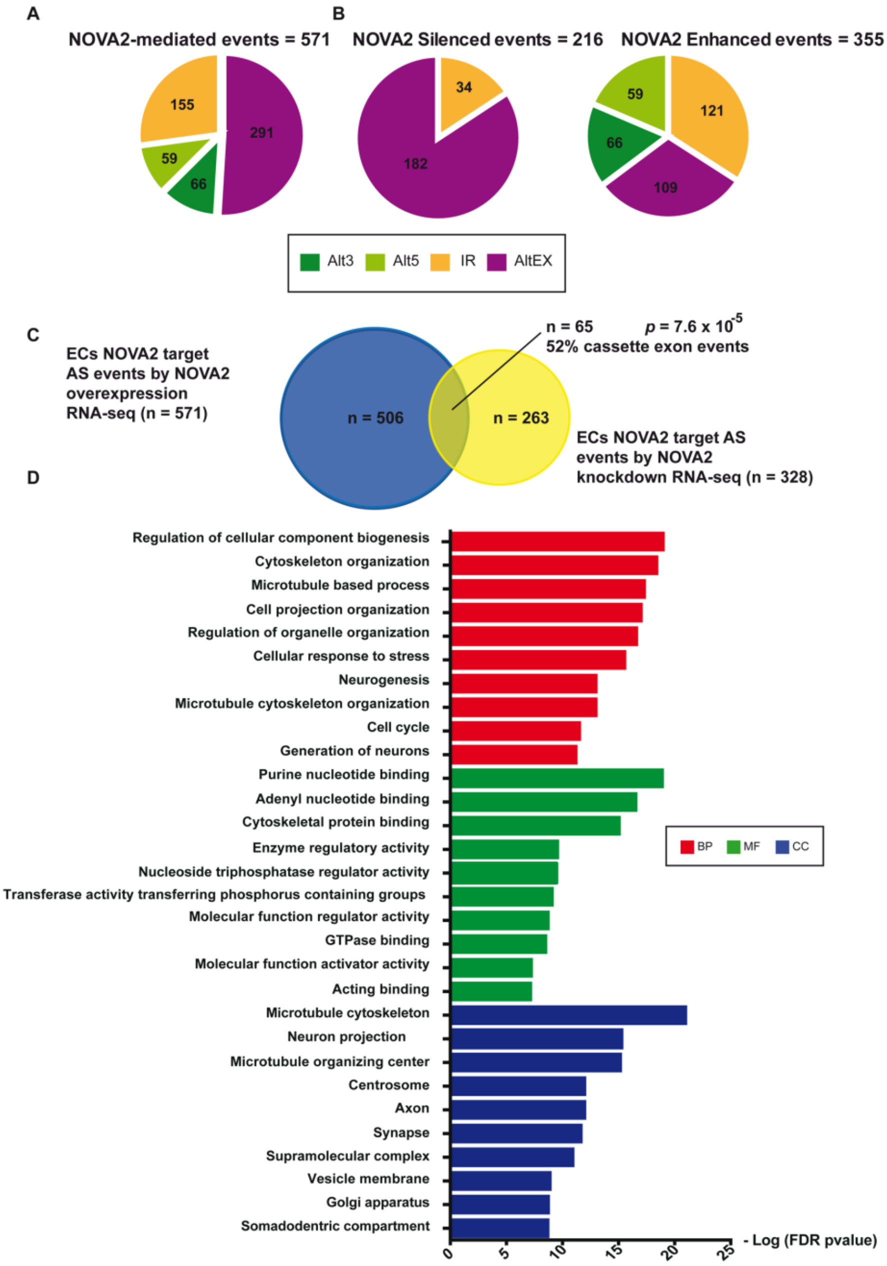

Figure S1. AS events modulated by NOVA2 overexpression in ECs. (**A**) NOVA2-mediated AS events classified according to the type of splicing mechanism: alternative use of a cassette exon (AltEx), intron retention (IR), alternative 5′ splice site (Alt5) and alternative 3′ splice site (Alt3). (**B**) Distribution of AS type subdivided in NOVA2 silenced events (left) and NOVA2-enhanced events (right). (**C**) Venn diagram showing the common AS events identified by RNA-Seq in NOVA2 overexpressing ECs (this work, blue circle) and previous published data in NOVA2 knockdown cells (yellow circle) [29]. The number (n) of identified genes is reported. (**D**) Gene ontology (GO) analysis of NOVA2-regulated genes using the MSigDB program. The first 10 terms of the “Biological Process” (BP in red), “Molecular Function” (MF in green) and “Cellular Component” (CC in blue) GO categories are indicated (sorted by FDR *p* value < 0.05 graphed as -Log).

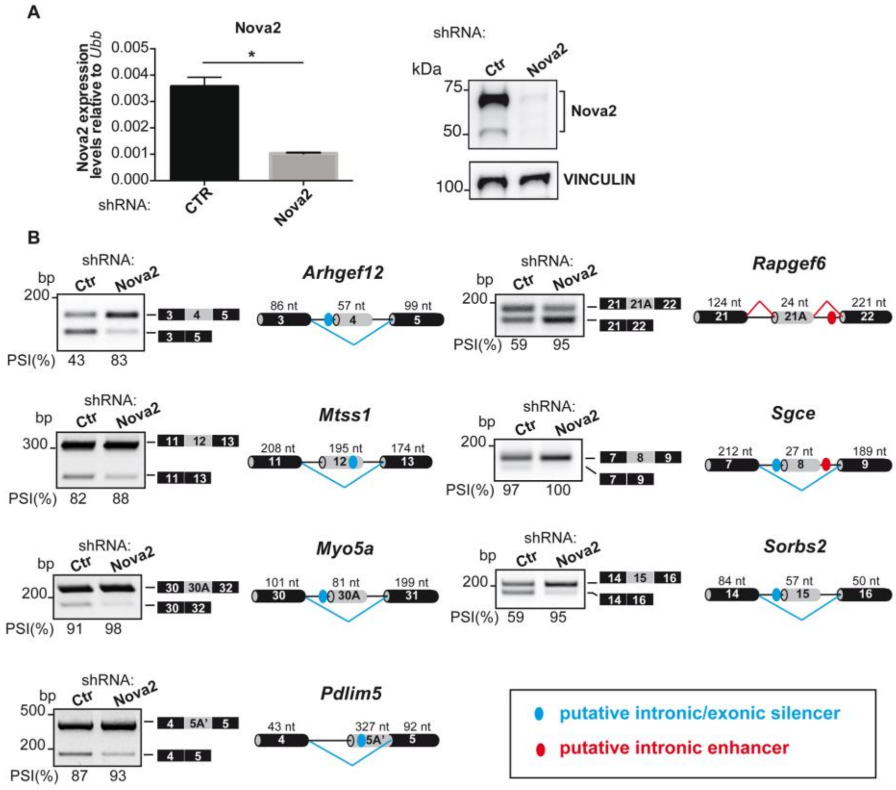

Figure S2. Validation of Nova2-mediated AS events in mouse Nova2 knockdown ECs. (**A**) Left: *Nova2* mRNA levels in mouse ECs (moEC) transduced with lentiviral vectors carrying control or *Nova2* shRNAs. Data represent the mean ± SEM (n =3 independent experiments). * $p \leq 0.05$. Unpaired Student's t-test. Right: NOVA2 immunoblotting in control and *Nova2* depleted moEC. (**B**) RT-PCR analysis of selected NOVA2 targets in moEC knockdown for *Nova2*. Transcripts generated from skipping/inclusion of the AS exon are represented near the corresponding RT-PCR bands and the percentages of exon inclusion (PSI) are indicated below each gel panel. For each AS event, the genomic region containing the AS exon and the flanking sequences are represented: grey boxes, AS exons; black boxes, constitutive exons; blue/red dots, YCAY clusters predicted to function as NOVA silencer/enhancer; blue/red lines, NOVA silenced/enhanced exon inclusion events.

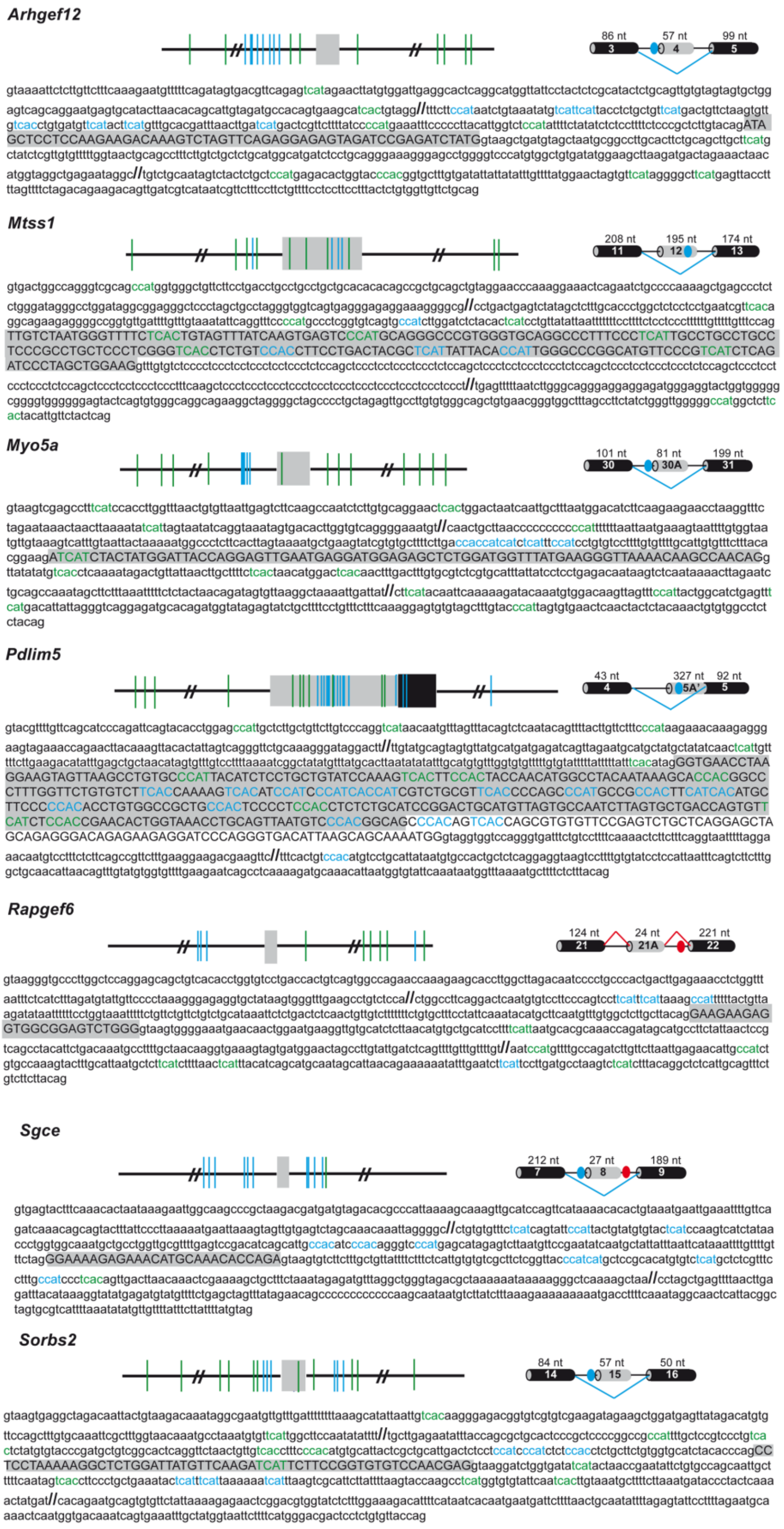
Arhgef12
86 nt
57 nt
99 nt
3
4
5
Mtss1
208 nt
195 nt
174 nt
11
12
13
Myo5a
101 nt
81 nt
199 nt
30
30A
31
Pdlim5
43 nt
327 nt
92 nt
4
5A'
5
Rapgef6
124 nt
24 nt
221 nt
21
21A
22
Sgce
212 nt
27 nt
189 nt
7
8
9
Sorbs2
84 nt
57 nt
50 nt
14
15
16

Figure S3. Putative Nova2 binding sites in novel Nova2 targets identified in mouse ECs. For each selected AS event, the sequence including the first 200 nt and the last 200 nt of the upstream intron, the AS exon (grey box), the first 200 nt and the last 200 nt of the downstream intron were analyzed with RBPmap (http://rbpmap.technion.ac.il). For *Pdlim5*, 200 nt downstream the second (distal) 3' splice site were considered. For each target, schematic representation of the NOVA binding sites (YCAY in which Y= pyrimidine) identified by RBPmap (blue lines and text), the mouse genomic sequences around the AS exons and the constitutive exons (black boxes) are reported. Green lines (and text) represent additional YCAY motifs conserved among mouse and human genes identified by manual inspection. Left: representation of putative NOVA enhancer (red dot) or silencer (light blue dot) elements, inferred from the position of YCAY clusters, and their evolutionary conservation between the mouse and human genes, are also present.

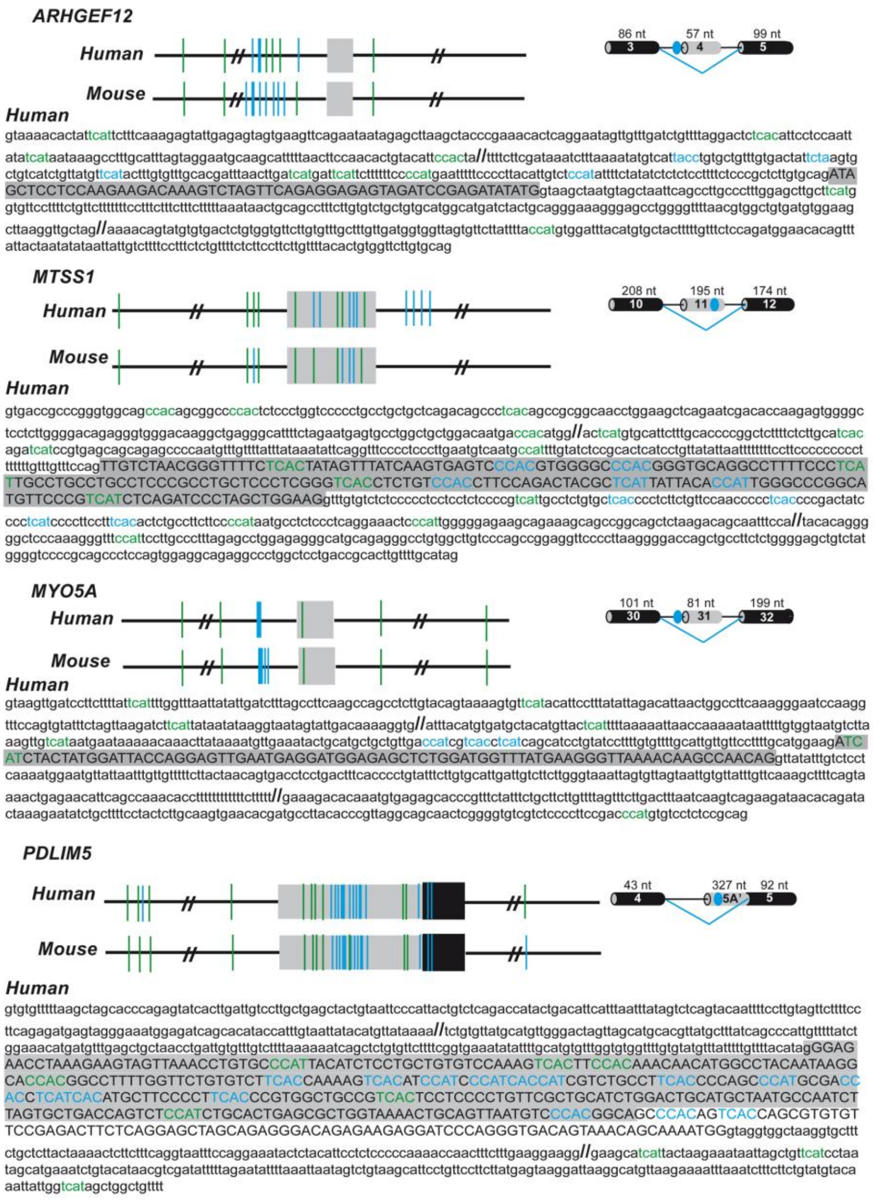
ARHGEF12
Human
Mouse
86 nt
57 nt
99 nt
3
4
5
MTSS1
208 nt
195 nt
174 nt
10
11
12
MYO5A
101 nt
81 nt
199 nt
30
31
32
PDLIM5
43 nt
327 nt
92 nt
4
5A'
5

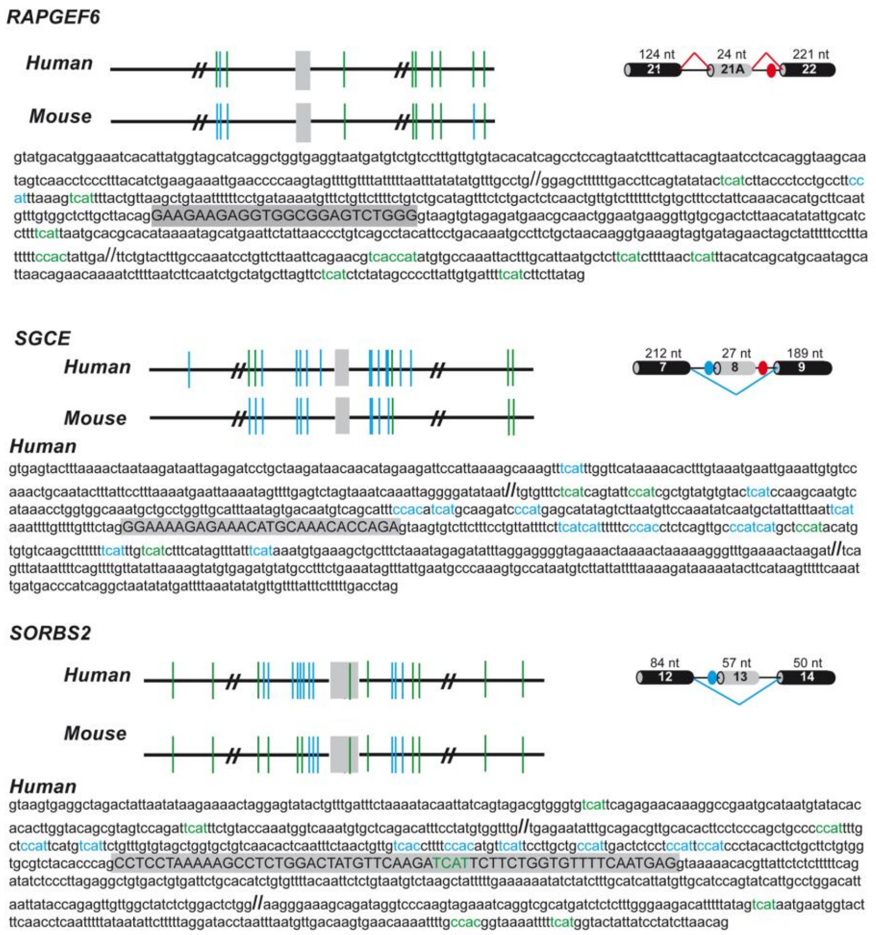

Figure S4. Putative NOVA2 binding sites in the corresponding human genes. For each selected AS event, the sequence including the first 200 nt and the last 200 nt of the upstream intron, the AS exon, the first 200 nt and the last 200 nt of the downstream intron were analyzed with RBPmap (http://rbpmap.technion.ac.il). For *PDLIM5*, 200 nt downstream the second (distal) 3′ splice site were considered. To define the position of putative NOVA enhancer (red dot) or silencer (light blue dot) elements represented in Figure 1, Figure 2 and Supplementary Figure S3, the presence of YCAY clusters and their evolutionary conservation between the mouse and human genes were considered. NOVA binding sites identified by RBPmap (blue lines) in mouse and human genomic sequences around AS exons. Green lines represent additional YCAY motifs conserved among mouse and hu-man genes identified by manual inspection.

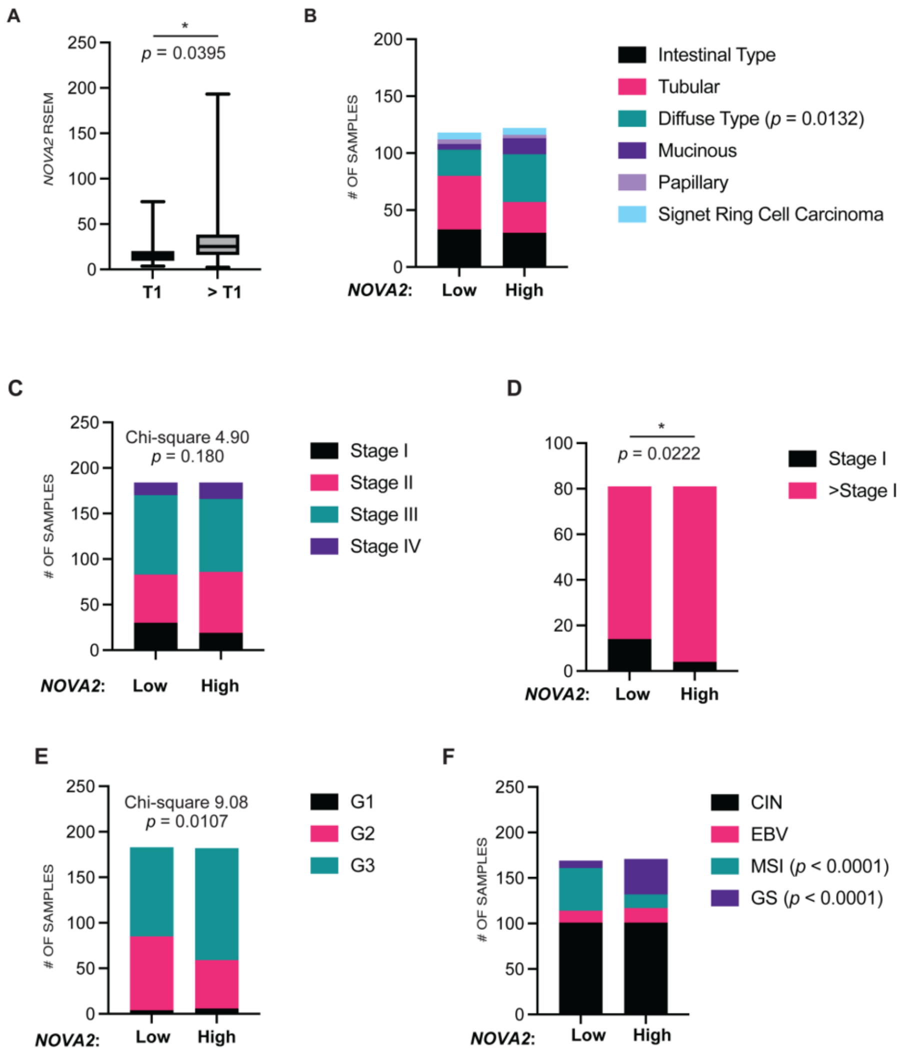

Figure S5. Clinico-pathological features of the TCGA-STAD patients according to *NOVA2* expression levels. (**A**) *NOVA2* mRNA expression levels (RSEM) in the TCGA-STAD dataset according to tumor size: T1; or >T1 (T2, T3, T4). Unpaired Student`s t-test. * $p < 0.05$. (**B**) Distribution of GC subtypes in the TCGA-STAD dataset according to *NOVA2* expression (cutoff: median). Fisher`s exact test. (**C**) GC tumor stages classification (I, II, III, and IV) in the above TCGA-STAD patients (Chi-Square test) and (**D**) in the TCGA-STAD intestinal subtype (* $p < 0.05$; Fisher`s exact test). (**E**) Tumor grade (G1, G2, and G3) classification of TCGA-STAD tumors stratified accordingly to *NOVA2* expression (cutoff: median). Chi-square test. (**F**) Molecular subtype distribution of GC tumors in low and high *NOVA2* expressing tumors (cutoff: median). CIN = chromosomal instability; EBV = Epstein–Barr virus-associated GC; MSI = microsatellite instability; GS = genomic stable. Fisher`s exact test to compare each subtype with all others.

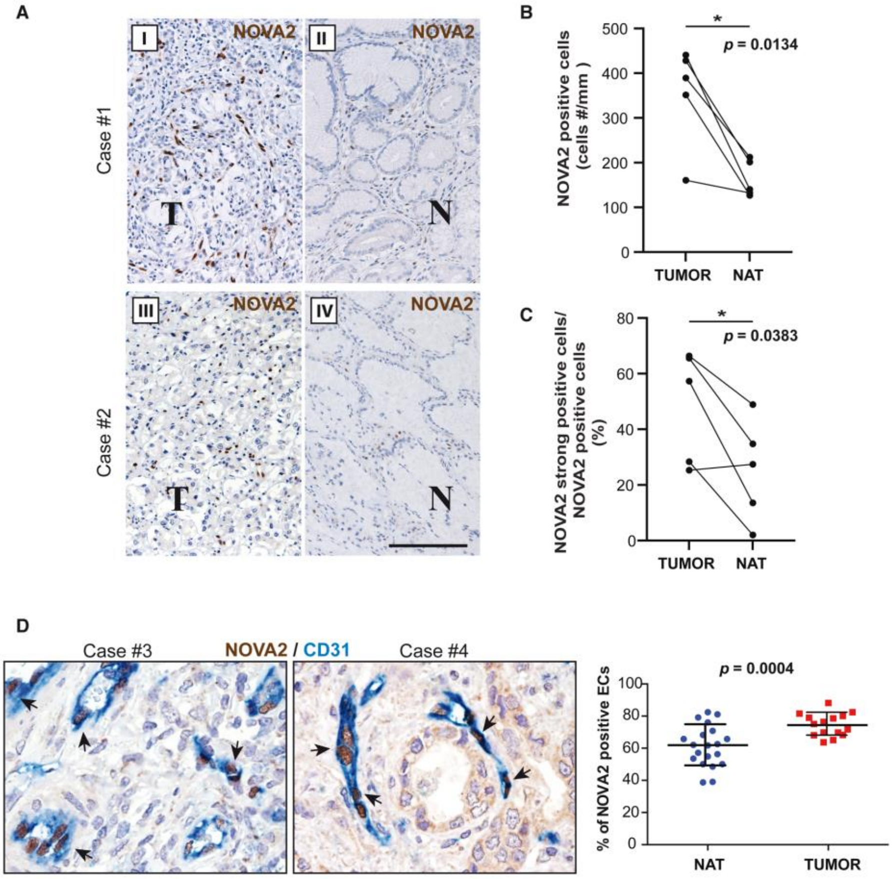

Figure S6. Expression of NOVA2 in GC and healthy gastric vessels. (**A**) IHC analysis of NOVA2 in an independent cohort of GC patients (n=5). Representative IHC images for NOVA2 staining in two cases of CG (I and III) and normal adjacent tissues (II and IV). Scale bar: 200 µm. (**B-C**) Quantification of NOVA2 positive cells (upper panel) and NOVA2 positive cells with strong IHC signal (lower panel) relative to total cell area and total NOVA2 positive cells, respectively, in tumor specimens (TUMOR, T) and normal adjacent tissue (NAT, N). (**D**) Left: representative images of NOVA2 and CD31 double staining of tumor and normal sections of GC patient's cohort (magnification: 400X). Arrows indicate ECs positive for both NOVA2 (blue) and CD31 (brown). Right: count of percentage of NOVA2 positive ECs on the total of ECs stained with the anti-CD31 antibody in tumor vs normal adjacent tissues of GC patients. Two-tailed Student's t-test with Welch's correction. Error bars indicate ± SEM. Exact *p* values (*p*) are indicated: * *p* value < 0.05.

Table S1. Information regarding the 571 AS events regulated by NOVA2 overexpression in mouse ECs. (**A**) "NOVA2 OE ECs RNA-Seq" GENE: gene name, the first column reports the name retrieved from RNA-Seq, the second column the up-to-date nomenclature. EVENT: vastDB identification code. COORD: coordinates relative to the regulated exon and referred to mm9 assembly. LENGHT: dimension in nucleotide of regulated AS event. FullCO: chromosome location: C1 donor, AS exon, C2 acceptor. Strand is "+" if C1 donor coordinate is smaller than C2 acceptor coordinate, and "-" otherwise. COMPLEX: Alt3/Alt5, alternative splice site acceptor/donor selection; IR, intron retention; S, C1, C2, C3, MIC ANN are cassette alternative exons. For each sample, reads count and quality score were reported (columns N-S and Z-AE); columns H-M and T-Y report reads count and their quality for control and *NOVA2* knockdown samples (from [22]). Last three columns provide splicing information as obtained from *vast-tools* (mean PSI of CTR and NOVA2-overexpressing samples). dPSI: difference in the percent splicing inclusion between overexpressing and control ECs. (**B**) "COMMON TARGETS NOVA2 OE shNOVA2" contains common AS events between NOVA2-overexpressing (this paper) and NOVA2-depleted ECs [29]. (**C**) "Direction of change AS events" illustrates the outcome of identified common NOVA2-mediated event (+ = NOVA2 promotes the splicing event; - = NOVA2 inhibits the splicing event).

Table S2. Gene ontologies (GO) and pathways enrichment of NOVA2 targets. Significantly enriched GO terms or pathways were obtained by using MSigDB (https://www.gsea-msigdb.org/gsea/msigdb/, accessed on 19 April 2023) [32,33]. The table collects all GO significant terms (FDR *p*-value ≤ 0.05) divided into "Biological Process" (**A**), "Molecular Function" (**B**), and "Cellular Component" (**C**). Significantly enriched pathways are collected in (**D**).

Table S3. Significant gastric cancer-associated genes. (**A**) Significant enrichment in genes upregulated upon *H. pylori* infection with MySignDB. (**B**) Enriched terms from Orphanet gene-phenotype database collected in Enrichr.

Table S4. NOVA2 expression and clinico-pathological data of gastric cancer patients. (**A**) "NOVA2 from TSVdb": Column A: Sample Identifier for TCGA samples, Column B-J: clinico-pathological features of cancer patients, including overall survival (os) in day; Column K-S: Expression values of NOVA2 gene and isoform data, exon, and junction-normalized read count data (in RSEM). (**B**) "NOVA2 from cBioPortal": Other clinical-pathological features (molecular subtype, stage, T-stage and grade) of TCGA patients; (**C**) "Oncomine Dataset Wang" and (**D**) "Oncomine Dataset DErricco": NOVA2 expression values of normal and tumor patients retrieved from Oncomine.

Table S5. Count of NOVA2 positive ECs of tumor and normal adjacent tissue of gastric cancer patients. For five different cases, three field for section (K1-K15) were counted for tumor regions while four field for section (N1-N20) were counted for normal adjacent tissues, calculating the percentage of NOVA2-positive (+) cells on the total of CD31-positive ECs.

Table S6. *RapGEF6* exon 21A in gastric cancer patients. (**A**) **"**RapGEF6 from TSVdb". Column A: Sample Identifier for TCGA samples, Column B-J: clinico-pathological features of patients, including overall survival (os) in day; Column K-S: Expression values of *RapGEF6* gene and isoform data, exon, and junction-normalized read count data (in RSEM). (**B**) "NOVA2 RapGEF6 exon 21A Correlation" Column A: Sample Identifier for TCGA samples, Column B: Clinical sample type (only primary tumors). Column C: Expression values (RSEM) of NOVA2 gene. Column D: Expression values (RSEM) of *RapGEF6* exon 21A (coordinates: chr5:130785716-130785739). (**C**) "RapGEF6 exon 21A and T stage" Column A: Clinical sample type (only primary tumors). Column B: Sample Identifier for TCGA samples, Column C: T (tumor) size (T1, T1A, T1B, T2, T2A. T2B, T3, T4) according to TNM classification. Column D: Expression values (RSEM) of *RapGEF6* exon21A (coordinates: chr5:130785716-130785739). (**D**) **"**RapGEF6 exon 21A+Type of tumor". Column A: Sample Identifier for TCGA samples, Column B: Clinical sample type (only primary tumors). Column C: Expression values (RSEM) of *RapGEF6* exon 21A (coordinates: chr5:130785716-130785739). Column D: GC histoypes. (**E**) "Survival curve RapGEF6 21A". Column A: Sample Identifier for TCGA samples. Column B: Expression values (RSEM) of *RapGEF6* exon 21A (coordinates: chr5:130785716-130785739). Column C: Overall Survival (os) status. Column D: Time (in months) STAD patients were still alive.

(**F**) "ARG signature NOVA2+ RAPGEF6 21A". Column A: Sample Identifier for TCGA samples, Column B: Clinical sample type (only primary tumors). Column C: Expression values (RSEM) of NOVA2 gene. Column D: Expression values (RSEM) of *RapGEF6* exon 21A (coordinates: chr5:130785716-130785739). Column E-W: Expression values (RSEM) of ARG genes. Column X: Calculated ARG signature.

Table S7. List of primers used in qPCR and RT-PCR experiments.

**Primers for PCR**	
**PRIMER NAME**	**SEQUENCE 5'-3'**
Arhgef12_mouse_exon3_F	TTTGAACCGAGAGTCACCAAC
Arhgef12_mouse_exon5_R	CCATTGTCGTCTTTCTGGATG
Mtss1_mouse_exon11_F	CCTTCCAGTCCAAGTCACCC
Mtss1_mouse_exon13_R	GGGTCCTCCCCCATTAGAGT
Myo5a_mouse_exon30_F	ACAAAGGTGAAATAGCACAAGCA
Myo5a_mouse_exon 31_R	GCTGTTGCCGGTTGTTTTCT
Pdlim5_mouse_exon4_F	CCAAGAGTGAGCCGGTTTCC
Pdlim5_mouse_exon6_R	CAGTGTCCTCAATCAGCCGT
Rapgef6_mouse_exon21_F	TGACTTCTGCCAACATGGAC
Rapgef6_mouse_exon22_R	ACCATATGCAGGTTCCCACT
Sgce_mouse_exon7_F	GGATTTCCTCGTTACCCTGGC
Sgce_mouse_exon9_R	ATCGAGCTGTGATGGACAAGC
Sorbs2_mouse_exon14_F	CGGGTCGCATAAATCCAGC
Sorbs2_mouse_exon17_R	CTAAAGTCACCGGCCATGCT
ARHGEF12_Human_exon3_F	TGAACCGAGAGTCACCAACA
ARHGEF12_Human_exon5_R	CGAAGACTGGATTGTCTCCA
MTSS1_Human_exon10_F	CAGTCCAAGTCACCATCCCC 4
MTSS1_Human_exon13_R	GTGGCAGCCGATACAGTCAT

MYO5A_Human_exon30_F	AGCACAAGCATACATTGGTTTGA
MYO5A_Human_exon32_R	TGCTGTCGGTTGTTCTCCTC
PDLIM5_Human_exon4_F	AGCATCTGCTGCACCCAAG
PDLIM5_Human_exon6_R	CTTGGACGCCAGTCTTCAGT
RAPGEF6_Human_exon21_F	TCTGCTAACATGGACCCAGC
RAPGEF6_Human_exon22_R	TGCCATTTGGGCATCCTCAT
SGCE_Human_exon7_F	GCAGTGGCACTGGTCCTTTTT
SGCE_Human_exon9_R	TGGACATGTCTCGAAGCTCCT
SORBS2_Human_exon12_F	AGAACTGGAGTTTGGACGCC
SORBS2_Human_exon15_R	GTGCTTGATCCTGGGAGGTC
**Primers for qPCR**	
NOVA2_Human_FOR	CAGCTTTATTGCCGAGAAGG
NOVA2_Human_REV	ACCCATGCTCCTGACTGTTC
Nova2_mouse_FOR	TGCTGTCCACAGCTTTATCG
Nova2_mouse_REV	GCTCCTCCCTTACCGATGAT
GAPDH_FOR	TCAAGAAGGTGGTGAAGCAGG
GAPDH_REV	ACCAGGAAATGAGCTTGACAAA
Ubb_FOR	CCGGCAAGCAGCTAGAAGAT
Ubb_REV	ATTGGGGCAAGTGGCTAGAG
